# Cyclosporine A but Not Corticosteroids Support Efficacy of *Ex Vivo* Expanded, Adoptively Transferred Human Tregs in GvHD

**DOI:** 10.3389/fimmu.2021.716629

**Published:** 2021-10-11

**Authors:** Sybille Landwehr-Kenzel, Anne Zobel, Isabela Schmitt-Knosalla, Anne Forke, Henrike Hoffmann, Michael Schmueck-Henneresse, Robert Klopfleisch, Hans-Dieter Volk, Petra Reinke

**Affiliations:** ^1^Department of Pediatric Respiratory Medicine, Immunology and Critical Care Medicine, Charité – Universitätsmedizin Berlin, Corporate Member of Freie Universität Berlin and Humboldt-Universität zu Berlin, Berlin, Germany; ^2^Berlin Institute of Health (BIH) at Charité – Universitätsmedizin Berlin, BIH-Center for Regenerative Therapies (BCRT), Berlin, Germany; ^3^ Berlin Center for Advanced Therapies (BeCAT), Charité - Universitätsmedizin Berlin, Corporate Member of Freie Universität Berlin and Humboldt-Universität zu Berlin, Berlin, Germany; ^4^ Institute of Medical Immunology, Charité – Universitätsmedizin Berlin, Corporate Member of Freie Universität Berlin and Humboldt-Universität zu Berlin, Berlin, Germany; ^5^Department of Veterinary Medicine, Institute of Veterinary Pathology, Freie Universität Berlin, Berlin, Germany; ^6^Department of Nephrology and Internal Intensive Care Medicine, Charité – Universitätsmedizin Berlin, Corporate Member of Freie Universität Berlin and Humboldt-Universität zu Berlin, Berlin, Germany

**Keywords:** transplantation, regulatory T-cells, adoptive cell therapy, tolerance, drug interaction, Graft-versus-Host Disease, immunosuppressive drugs

## Abstract

Reshaping the immune balance by adoptive transfer of regulatory T-cells (Tregs) has emerged as a promising strategy to combat undesired immune reactions, including in Graft-versus-Host Disease (GvHD), which is the most lethal non-relapse complication of allogeneic hematopoietic stem cell transplantation. Currently however, little is known about the potentially inhibitory *in vivo* effects of conventional immunosuppressive drugs, which are routinely used to treat GvHD, on adoptively transferred Tregs. Here we demonstrate drug-specific effects of the conventional immunosuppressive drugs Cyclosporine A, Mycophenolate mofetil and methylprednisolone on adoptively transferred Tregs in a humanized NOD/SCID/IL2Rgamma-/- GvHD mouse model. The clinical course of GvHD and *postmortem* organ histology, including cellular organ infiltration, showed that co-administration of Cyclosporine A and Tregs is highly beneficial as it enhanced Treg accumulation at inflammatory sites like lung and liver. Similarly, co-administration of Mycophenolate mofetil and Tregs improved clinical signs of GvHD. In contrast, co-administration of methylprednisolone and Tregs resulted in reduced Treg recruitment to inflammatory sites and the fast deterioration of some animals. Consequently, when clinical trials investigating safety and efficacy of adjunctive Treg therapy in GvHD are designed, we suggest co-administering Cyclosporine A, whereas high doses of glucocorticosteroids should be avoided.

## Introduction

Allogeneic hematopoietic stem cell transplantation (HSCT) is a curative treatment option for many patients with malignant and non-malignant diseases of the hematopoietic system. Graft-versus Host-Disease (GvHD) however, still constitutes one of the most lethal post-transplant complications of HSCT (1). While pharmacologic immunosuppression has dramatically improved short-term outcomes, long-term morbidity and mortality is still poor in patients suffering from GvHD. Despite the beneficial factor of graft-versus leukemia effects in patients with malignant underlying disease, transplantation related mortality rises from 5-13% in patients without GvHD to 51-75% in patients suffering from any form of GvHD (2). The current paradigm holds that GvHD is primarily a T-cell mediated disease caused by donor effector-T-lymphocytes recognizing allogenic structures as non-self in the host, and thereby eliciting their proinflammatory and cytolytic program. Our understanding of immunoregulatory mechanisms profoundly advanced with the identification of thymic derived regulatory T-cells (tTregs) as physiologic counterplayers of effector T-cells and critical mediators of immune homeostasis after T-cell activation by self- and non-self structures (3). tTregs are characterized by high expression of CD25 and the transcription factor FoxP3, and characteristically low expression of CD127. All three proteins are established as markers for Treg diagnostics. A numeric loss of tTregs is associated not only with a higher incidence and severity of GvHD (4–8) but also with autoimmune diseases (9–11). In contrast, therapeutic benefits can be achieved by enrichment of circulating tTregs after HSCT (12–19) and in the context of solid organ transplantation (20–22) and autoimmune diseases such as hepatitis C vasculitis (23), systemic lupus erythematosus or Type 1 diabetes (24–26).

Recent technological developments have enabled the purification and *ex vivo* expansion of Tregs on a large scale and under clinical grade conditions. This has allowed the translation of adoptive tTreg transfer from animal models to clinical protocols. In order to investigate safety and efficacy, Tregs will have to be administered as adjunctive therapy in addition to current state of the art protocols in early clinical trial phases. Therefore, in the context of HSCT, Treg transfer will have to be combined with the conventional immunosuppressive drugs (cISD) Cyclosporine A (CSA), mycophenolate mofetil (MMF) and prednisolone or methylprednisolone (MP) as components of prophylactic as well as first-, second- and third-line treatment regimens.

The question of “if” and “how” cISD influence the function and frequency of tTregs *in vivo* has previously been addressed, but some of the data are contradictory. Furthermore, there is little data available describing the effects of these drugs on the *in vivo* fate of *ex vivo* manipulated, adoptively transferred tTregs (27). Direct comparison of individual cISD in a clinical setting is difficult, since standard immunosuppressive regimens in HSCT but also for solid organ transplantation are comprised of a combination of at least two agents. Therefore, most data investigating individual drug-specific effects were generated by *in vitro* or animal experiments. The prevailing opinion assigns a beneficial effect of mTOR (Rapamycin, Everolimus) (28) and histone deacetylase inhibition (29, 30) but also low-dose Interleukin-2 (18, 23, 31–36) and anti-thymocyte globulin (37, 38) to tTreg maturation, function and survival. In contrast, the calcineurin inhibitors (CNI) CSA and Tacrolimus (20, 27, 28, 39–48), and blockage of CTLA-4 (49) and Interleukin-2 (50) seems to hamper both tTreg development and function. Even less consistent data exists on the effect of complement inhibitors, steroids (51–56) and MMF (43, 57). The effect of cISD on *ex vivo* expanded and adoptively transferred tTregs are just now starting to receive attention, however, study designs and results are diverse.

Therefore, the aim of this study was to determine the drug-specific influence of the standard prophylactic and therapeutic first-, second- and third-line drugs, CSA, MMF and MP used in GvHD therapy, on adoptively transferred Tregs. In order to investigate the pharmacological effects of cISD at the cellular level *in vivo*, as well as to obtain a deeper understanding of beneficial combinations of cISD with adjunctive Treg therapy in the clinical setting of GvHD, a humanized GvHD mouse model was employed. Insights obtained from this work will pave the way for rational design of prospective Phase I and II clinical trials investigating safety and efficacy of adjunctive Treg therapy in patients with acute and chronic GvHD.

## Materials and Methods

### Cell Isolation

Peripheral blood mononuclear cells (PBMC) were isolated from freshly collected blood of healthy volunteers or from buffy coats by Ficoll density-gradient centrifugation (PAA Laboratories, Pasching, Austria). Treg isolation was performed using the CD4+CD25+ Treg isolation kit (Miltenyi Biotech, Bergisch Gladbach, Germany) according to the manufacturer’s recommendations. This study was approved by the Charité-Universitätsmedizin Berlin ethics committee (Institutional Review Board) and all blood donors provided written informed consent.

### Treg Expansion

Freshly isolated Tregs were polyclonally expanded as described previously (13). Briefly, Tregs were cultured in X-VIVO15 medium (Lonza, Basel, Switzerland) containing 10% serum, 500 U/mL human recombinant IL-2 (Chiron Behring, Marburg, Germany) and 100 nM rapamycin (Sigma–Aldrich, St. Louis, MO) and stimulated with CD3/CD28 Treg expansion beads (Miltenyi Biotech). Cells were restimulated every 3-4 days. After 3-weeks expansion time tTreg product purity was assessed by specific staining of CD3-APC750 (Clone UCHT1), CD4-APC (Clone 13B8.2), CD8-A700 (Clone B9.11), CD25-PeCy7 (Clone B1.49.9) from Beckman Coulter, Krefeld Germany) and FoxP3-A488 (Clone 259D/C7, Beckton Dickinson, Heidelberg, Germany). Mean Treg purity after isolation was 80% for CD3^+^CD4^+^ and 60% for CD3^+^CD4^+^CD25^high^ cells. After isolation Tregs were expanded in cell culture medium supplemented with IL-2 and rapamycin in order to promote Treg expansion and suppress expansion of non-Treg cells. The final Treg products consisted of ~90% CD3^+^CD4^+^ T-cells with a frequency of 94% CD25^high^FoxP3^+^ Tregs.

### Humanized Animal Experiments for Xenogenic GVHD

**Figure 1:**
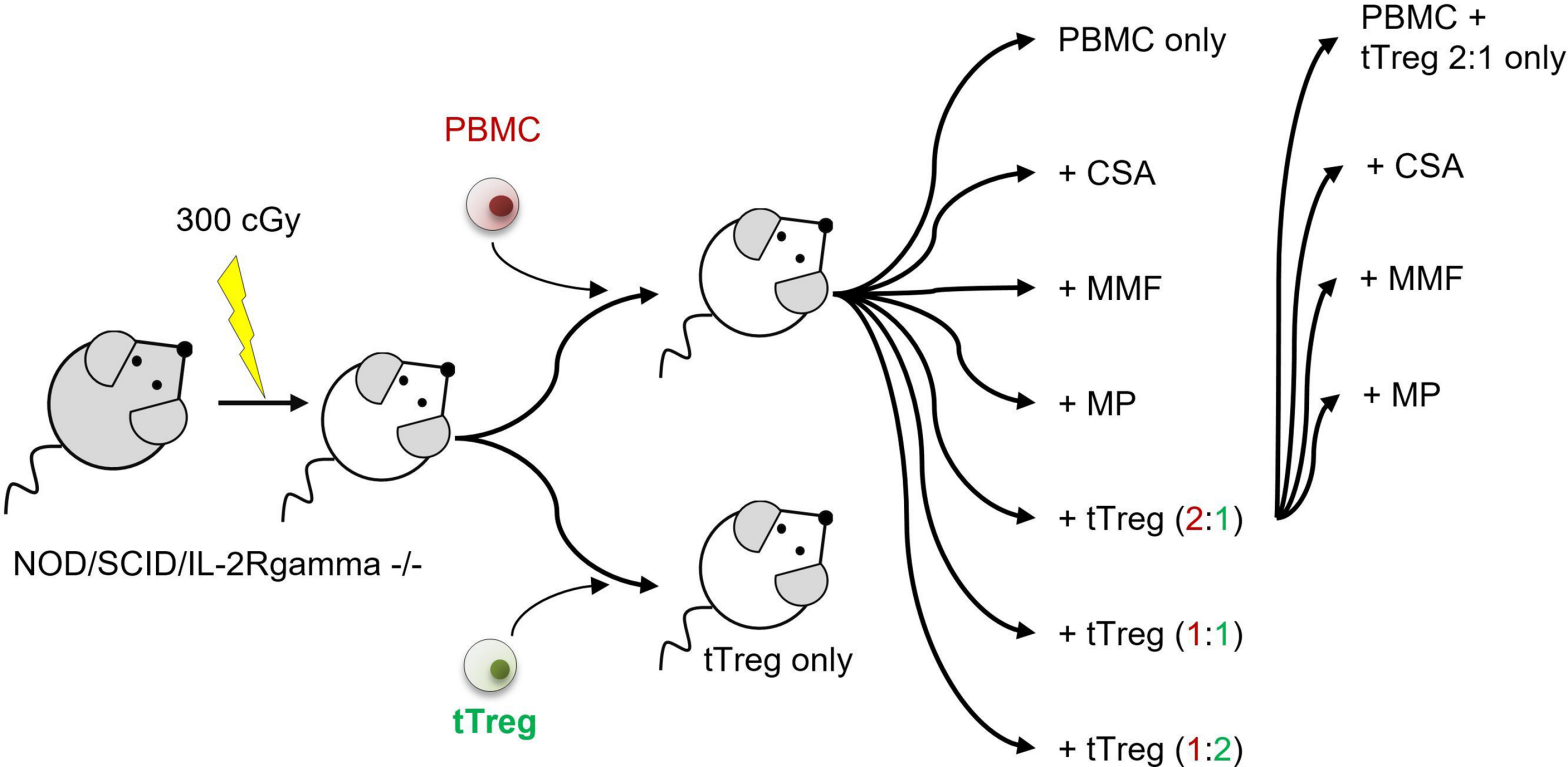
Experimental setup. NOD/SCID/IL-2Rgamma^-^/^-^ mice were sub-lethally radiated with 300 cGy (day -1). Xenogenic GvHD was induced by intravenous application of 3x10^6^ human PBMCs. GvHD control mice did not receive further therapy. tTreg control mice received 3x10^6^ *ex vivo* expanded tTregs. If indicated tTregs were simultaneously administered with PBMCs at a dosage of 1.5x10^6^ (PBMC:tTreg ration of 2:1), 3x10^6^ (PBMC:tTreg ratio of 1:1) or 6x10^6^ (PBMC:tTreg ratio of 1:2). Mice that received either PBMCs alone or in combination with tTregs were further treated daily with the cISD CSA (4 mg/kg/d s.c.), MMF (0.5 mg/d p.o) or MP (20 mg/kg/d i.p.) starting day +1 after cell administration. Animals were monitored daily for body weight and clinical signs of GvHD (behavior, fur alterations, skin inflammation, hunched back). Liver, spleen, and lungs were collected on day +35 or when clinical signs required euthanasia.

The adult offspring of NOD/SCID/IL-2Rgamma^-^/^-^ mice, originally purchased from Charles River Laboratories (Research Models and Services, Sulzfeld, Germany), were used according to the approval by the Landesamt für Gesundheit und Soziales Berlin (G0483/09). Mice were maintained under specific pathogen-free conditions. Xenogenic GvHD was induced, after sublethal radiation (300 cGy), by single intravenous injection of 3x10^6^ human PBMCs/mouse. *Ex vivo* expanded human tTregs, that originate from the same donor as the PBMCs used to induce GvHD, were administered i.v. at a single dose of 1.5x10^6^, 3x10^6^, or 6x10^6^, equivalent to a PBMC:tTreg ratio of 2:1, 1:1 and 1:2. Additionally 1.5x10^6^ tTregs were combined with each of the cISD. Conventional immunosuppressive therapy was administered daily from day+1 until mice were sacrificed on day+35 or sacrificed due to progressive disease: Cyclosporine A 4 mg/kg s.c. (Novartis Pharma GmbH, Nürnberg, Germany), Mycophenolate Mofetil 0.5 mg p.o. (MMF, CellCept^®^ 1g/5ml Roche Pharma, Grenzach-Wyhlen Germany), Methylprednisolone 20 mg/kg/d i.p. daily (MP, Urbason ^®^ solubile 32 mg Sanofi Aventis Deutschland GmbH, Frankfurt am Main, Germany). cISD dose finding was based on our own experience (20) and previously published data (58–60) in order to mimic the clinical situation with serum levels similar to target levels used in human patients. During the experimental setup we aimed for equal distribution of male and female animals in all groups (**Supplementary Figure S1**). The first group included mice that received no form of therapy after the PBMC administration. To further control for the effect of tTregs alone, tTregs were administered to a second control group without prior administration of PBMCs. Animals were monitored daily for body weight and clinical signs of GvHD (behavior, fur alterations, skin inflammation, hunched back, **Supplementary Table ST1**) and scored as either no GvHD (0); mild GvHD (1 – 1.5), moderate GvHD (2 – 2.5) or severe GvHD (3). Livers, lungs, spleens and intestines were collected on day+35 or when clinical signs required euthanasia. Euthanasia due to a poor clinical condition was performed when body weight decreased by >20% of initial body weight or at a clinical score of 3. Details of experimental setup are depicted in **Figure 1** and days of analysis further listed in **Table 1**.

**Table 1:** Summary of experimental setup.

Group	Total number of animals	Number of independent experiments	Survival in days
			(=Days of organ analysis)
PBMC alone	6	3	18, 18, 24, 27, 29, 29
tTreg alone	3	2	35, 35, 35
PBMC:tTreg 2:1	6	3	15, 20, 26, 33, 34, 35
PBMC:tTreg 1:1	7	3	27, 29,31, 32, 35, 35, 35
PBMC:tTreg 1:2	5	2	22, 30, 25, 35, 35
PBMC + CSA	5	3	35, 35, 35, 35, 35
PBMC + MMF	5	3	10, 30, 35, 35, 35
PBMC + MP	5	4	35, 35, 35, 35, 35
PBMC + CSA + tTreg	5	2	35, 35, 35, 35, 35
PBMC + MMF + tTreg	6	3	31, 32, 35, 35, 35, 25
PBMC + MP + tTreg	6	3	24, 25, 31, 35, 35, 35

Animals were assigned to 10 treatment groups, including mice who received PBMCs alone, PBMCs plus Treg monotherapy at a PBMC:tTreg ratio of 1:2, 1:1 and 1:2, PBMCs plus cISD CSA (4 mg/kg/d s.c.), MMF (0.5 mg/d p.o) or MP (20 mg/kg/d i.p.) or PBMCs plus co-administration of tTregs and cISD as listed in column 1.

The total numbers of animals per group and the numbers of individual experiments per group are listed in the second and third column, respectively. Days of survival are listed for every mouse of each group in column four. All organs were processed for analysis directly after harvesting.

### Organ Flow-Cytometry

After animals were sacrificed their organs were collected, divided and either fixed in 5% formalin for histologic analysis or resuspended in saline for direct organ flow cytometry. To isolate cells for FACS analysis, organs were mechanically disrupted and further digested by DNAse and collagenase (Sigma Aldrich, Germany). Subsequently, erythrocytes were lysed and remaining cells were washed in PBS and culture medium. Lung sample cells were flushed through 100 µm and 70 µm pore sized filters before staining. Hepatic samples were centrifuged at 50 xg for 3 minutes after cellular disruption and collagen digestion. Most hepatocytes sedimented whilst lymphocytes remained in the supernatant. The supernatant was collected and spun at 200xg for 2 minutes. The lymphocytes sedimented and were subsequently collected. Lymphocytes were additionally enriched by density centrifugation using Lymphocyte Separation Medium (LSM1077, PAA Laboratories, Pasching, Austria) before erythrocytes were lysed and cells subjected to the routine staining procedure. Antibodies were titrated to establish optimal staining conditions before use: CD3-APC-A750 (Clone UCHT1), CD4-APC (Clone 13B8.2), CD8-APC-A700 (Clone B9.11), CD19-ECD (Clone J3-119), CD25-PeCy7 (Clone B1.49.9), CD45-PacificBlue (Clone J33), CD56-PE (Clone N901), CD127-APC-A700 (Clone R34.34) from Beckman Coulter, Krefeld Germany; CD45 mouse-PerCP-Cy5.5 (Clone 30-F11, Biolegend, Germany); L/D PacificOrange (Life Technologies GmbH, Darmstadt, Germany); FoxP3-A488 (Clone 259D/c7), Ki67-PerCP-Cy5.5 (Clone B56) from Beckton Dickinson, Heidelberg, Germany. Flow cytometry was performed using Beckman Coulter NAVIOS cytometer. Data were analyzed using Kaluza^®^ software (Beckman Coulter). For staining, up to 2x10^6^ cells were suspended in 100 µl FACS buffer and unspecific Fc receptors were blocked using FcR blocking reagent (Miltenyi Biotech GmbH, Germany). Extracellular staining was performed as indicated. Cells were fixed and permeabilized using FoxP3 staining buffer set purchased from eBioscience, San Diego. Subsequently, cells were intracellularly stained, washed and analyzed by flow cytometry.

### Histology

Tissue samples of liver, lung, spleen, and kidney were fixed in 5% formalin, embedded in paraffin, and cut into 5 µm-thick slices. Slices were dewaxed and stained with hematoxylin/eosin. For microscopy an Olympus BX41 (Olympus, Hamburg, Germany) microscope with a 20-fold magnification lens was used. The grade of infiltration of human cells was calculated based on a semi-quantitative scoring system. The scoring system ranged from 0 (no); 1 (mild); 2 (moderate) up to 3 (massive) infiltration by inflammatory cells.

### Statistics

Unless stated otherwise, data are presented as scatter plots showing individual values and means ± SEM. Statistical significance was determined by Kaplan-Meier analysis and Log-rank test (Mantel-Cox-Test) or One-Way-ANOVA with Tukey correction. Significance levels are shown as indicated in the legends.

### Data Sharing

Most of the data are presented in the manuscript text, figures, and legends. Any additional data will be made available upon request by the corresponding author (sybille.landwehr-kenzel(at)charite.de).

## Results

### Co-Administration of CSA or MMF With Adoptive Treg Transfer Improves the Clinical Course of GvHD

**Figure 2:**
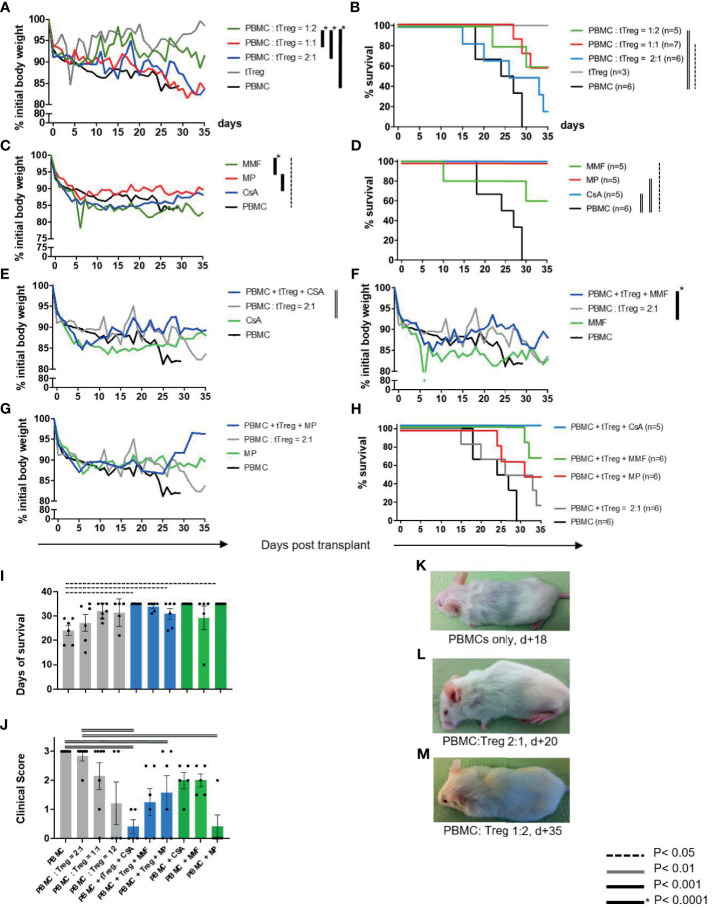
Clinical course and survival rates. As described in **Figure 1** GvHD was induced in NOD/SCID/IL-2Rgamma -/- mice by infusion of 3x10^6^ human PBMC [GvHD control, **(A–M)**]. tTreg control mice received *ex vivo* expanded human tTreg alone (3x10^6^) but no PBMCs [tTreg, **(A, B)**]. **(A, B)** Mice were treated with escalating doses of *ex vivo* expanded tTregs at a ratio of 2:1 (n=6), 1:1 (n=7) or 1:2 (n=5). **(C, D)** Alternatively, mice were treated with the cISD CSA (4 mg/kg s.c.), MMF (0.5 mg p. o.) or MP (20 mg/kg i.p.) (n=5) alone. **(E–H)** After GvHD induction by PBMCs mice were treated with tTregs in combination with CSA **(E, H)**, MMF **(F, H)** or MP **(G, H)**. **(J)** Clinical assessment further included clinical signs of GvHD (behavior, fur alterations, skin inflammation, hunched back, **Supplementary Table ST1**) and was quantified as either no GvHD (0); mild GvHD (1 – 1.5), moderate GvHD (2 – 2.5) or severe GvHD (3). **(K–M)** Representative images demonstrating the clinical condition of mice treated with no [**(K)**, d+18], low-dose [**(L)**, d+20] or high dose [**(M)**, d+35] tTregs are shown. Depicted are mean weight curves as percentage of initial body weight **(A, C, E–G)** and Kaplan-Meier-Survival curves **(B, D, H)**. Individual body weight values are depicted in the **Supplementary Figure S2**. To support survival curves, days of survival are further listed in **Table 1**. Statistical analysis of weight curves was performed as One-Way-ANOVA with Tukey correction for multiple comparison. Survival data were statistically analyzed using Log-Rank (Mantel-Cox) test. CSA, Cyclosporine A; MMF, Mycophenolate Mofetil; MP, Methylprednisolone.

To investigate the clinical effect of conventional immunosuppressive drugs on adjunctive tTreg therapy, GvHD was induced in NOD/SCID/IL-2Rgamma-/- mice by infusion of 3x10^6^ human peripheral blood mononuclear cells (PBMCs) (**Figure 1**, **Table 1** and **Figures 2A–M**). Severe GvHD, substantial weight loss and a higher GvHD score were observed in all mice (**Figures 2A, B, J**). The timing of clinical GvHD manifestation resembled the clinical course known from human HSCT, with disease onset usually occurring during the first 15 days after transplantation. Mice showed signs of GvHD such as hunched posture, scurfy hair and overall reduced general condition. One control group received only tTregs in the absence of PBMCs (**Figures 2A, B**). These mice did not show clinical signs of GvHD (**Figures 2A, B**). Following PBMC-mediated GvHD induction mice were treated once with escalating doses of *ex vivo* expanded tTregs in the absence of immunosuppressive drugs. Remarkably, monotherapy with tTregs led to a statistically significant dose-dependent reduction in weight loss (**Figure 2A**) and reduced GvHD activity (**Figure 2J**). In line, overall survival was significantly improved by higher doses of tTregs (**Figures 2B, I, J, K–M** and **Table 1**). Alternatively, mice were treated daily with the cISD CSA (4 mg/kg s.c.), MMF (0.5 mg p.o.) or Methylprednisolone (MP, 20 mg/kg i.p.) (**Figures 2C, D**). cISD dosages were based on previously published data in order to reach target serum levels similar to target trough levels in the clinical setting (20, 58–60). To investigate the effects of co-administered cISD on adoptively transferred tTregs, the lowest tTreg dose (PBMC: tTreg = 2:1) was combined with CSA, MMF or MP (**Figures 2A–J**). Mice that received any of the cISD alone suffered from substantial weight loss and in case of MMF monotherapy, showed partially reduced survival (**Figures 2C, D, I, J** and **Supplementary Figure S2**). Despite their poor clinical condition, some mice receiving CSA or MMF alone did not fulfill the endpoint criteria for euthanasia (**Figures 2D, I, J**). Their prolonged survival was associated with a lower clinical GvHD score (**Figures 2I, J**). CSA or MMF administration in addition to tTreg therapy, substantially improved the clinical course of GvHD (**Figures 2E, F, H–J** and **Table 1**). In contrast, co-administration of tTreg and MP was associated with fast deterioration of all animals and a poor clinical outcome (**Figures 2G, H-J**). Most importantly, combined therapy with tTreg/CSA was clinically more effective than any other treatment approaches investigated (**Figures 2A–J** and **Supplementary Figure S2**), resulting in minimal GvHD activity (**Figure 2H**) and 100% survival (**Figures 2H, I**). In summary, tTreg monotherapy ameliorated the clinical signs of GvHD and improved overall survival, whilst co-administration of CSA or MMF in addition to tTregs significantly improved the clinical outcome, even when combined with the lowest investigated tTregs dose.

### Combined Treatment of tTreg Therapy With CSA Reduces Lymphocytic Infiltration and Tissue Inflammation

**Figure 3:**
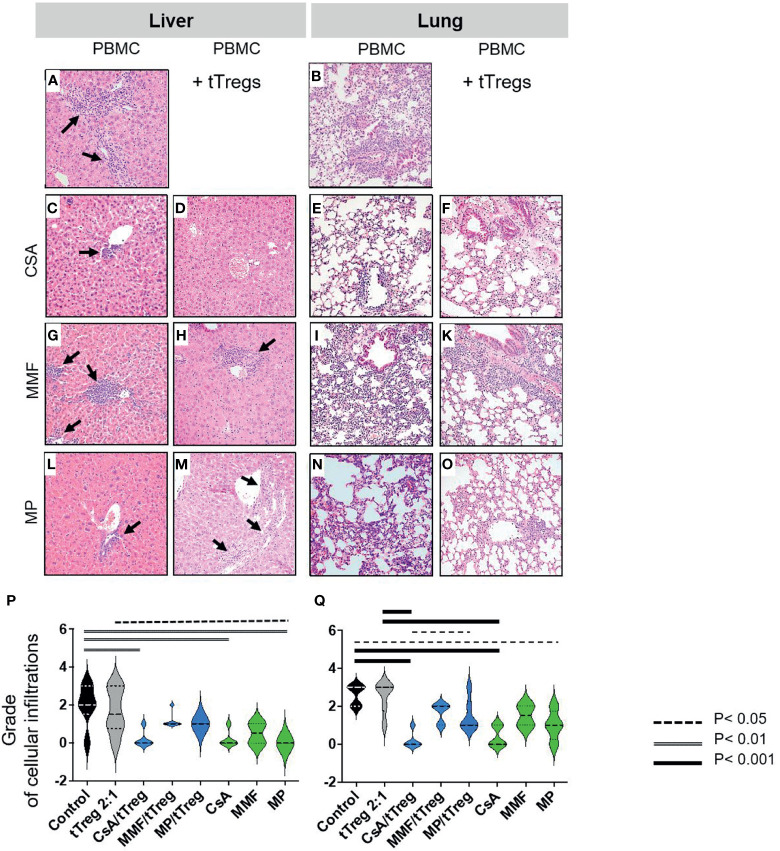
Histopathologic changes of liver and lung. Liver and lungs of mice described in **Figure 1** were stained with Hematoxylin/Eosin (HE). Depicted are **(A–O)** representative images from liver and lung samples at 20-fold magnification and **(A, B)** moderate to high-grade cellular infiltration and inflammation in liver and lungs of animals that received PBMCs but no GvHD therapy. **(A)** Liver shows significant bile duct damage, grade 3 lobular inflammation with dense lymphocyte infiltration and lymphocyte ballooning (arrows). **(B)** Lung tissue shows massive intra-alveolar and intraepithelial lymphocyte infiltration with inflammation of the pulmonary epithelium and apoptotic bodies as well as loss of alveolar tissue architecture. **(C–F)** Only mild lymphocyte infiltration and almost complete resolution of inflammation with preserved tissue architecture in both liver **(C, D)** and lung **(E, F)** were observed in mice treated with CSA **(C, E)** or CSA + Tregs **(D, F)**. **(G, H)** Significant reduction of hepatic lymphocyte infiltration and inflammation after MMF monotherapy; moderate infiltration of lymphocytes and signs of inflammation in mice which received combined MMF/tTreg therapy with preserved hepatic tissue architecture. **(I–K)** Only partial reduction of lymphocyte infiltration and inflammation in lung of mice which received MMF alone or in combination with tTregs. **(I)** Dense lymphocyte infiltration leading to diffuse alveolar damage and septal edema. **(K)** Alveolar architecture partially preserved after MMF/tTreg co-administration. **(L–O)** Significant reduction of cellular infiltration and preserved tissue architecture in both liver and lung upon treatment by MP alone or in combination with tTregs. **(P, Q)** shows quantification of lymphocyte infiltration of liver **(P)** and lung **(Q)**. Statistical analysis was performed as One-Way-ANOVA with Tukey correction for multiple comparisons. Statistical significance levels are indicated as p < 0.05 (dotted line), p < 0.01 (dashed line), p < 0.001 (bold line), p < 0.0001 (solid line with asterix). CSA, Cyclosporine A; MMF, Mycophenolate Mofetil; MP, Methylprednisolone.

Histopathologic organ morphology was assessed at the time of euthanasia in liver, lungs (**Figure 3**) and the intestine by microscopic analysis of tissue architecture, lymphocyte infiltration and signs of acute inflammation (**Figures 3A–Q**). To histologically semi-quantify these results, human CD3^+^ cells were stained and quantified in all samples of **(P)** livers and **(Q)** lungs. In line with previously published data (61–63) indicating that the lack of chemotherapeutic conditioning protects from intestinal GvHD, we did not find intestinal infiltrates or alternative signs of GvHD (**Supplementary Figure S3**). Accordingly, intestinal samples were not further analyzed here. In contrast, both the liver and lungs of animals that received PBMCs but not GvHD therapy showed moderate to high grade signs of GvHD (**Figures 3A, B**). In particular, we found hepatic bile duct damage with dense lymphocyte infiltration, inflammation and lymphocyte ballooning (**Figure 3A**). Lung pathology showed a significant loss of alveolar tissue architecture and was dominated by massive intra-alveolar and intraepithelial lymphocyte infiltration with inflammation of the pulmonary epithelium and apoptotic bodies (**Figure 3B**). Co-administration of CSA and tTregs (**Figures 3D, F**), and to a slightly lesser extent CSA monotherapy (**Figures 3C, E**), significantly reduced the histopathological grade of GvHD (**Figures 3P, Q**). This effect was characterized by preserved tissue architecture, mild lymphocyte infiltration and almost complete resolution of inflammation in both the liver and lung. Both MMF and MP administered as monotherapy or combined with tTregs only partially reduced lymphocyte infiltration but ameliorated signs of inflammation and largely preserved organ architecture (**Figures 3G–K**). In summary, therapy with both MMF and MP alone or in combination with tTregs was inferior to CSA and CSA/tTreg therapy. Accordingly, the beneficial effect of tTreg therapy on clinical parameters as described in **Figure 2** correlated with reduced lymphocytic organ infiltration.

**Figure 4:**
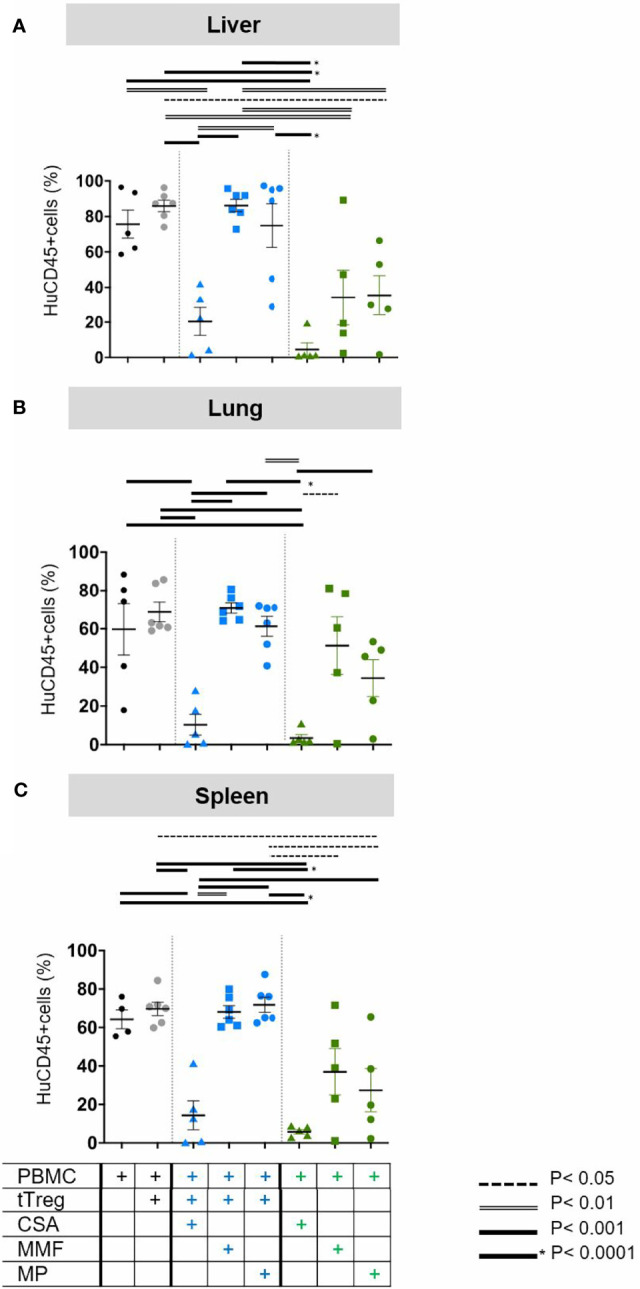
Quantification of human leukocyte infiltration. As described in **Figure 1** GvHD was induced by 3x10^6^ human PBMCs in NOD/SCID/IL2Rgamma -/- mice. Mice were subsequently treated with (i) the individual cISD (green symbols), CSA (4 mg/kg s.c., green triangles), MMF (0.5 mg p.o., green squares) or MP (20 mg/kg i.p., green dots) (n=5) alone (n=5), (ii) with *ex vivo* expanded tTregs at a PBMC:tTreg ratio of 2:1 (n=6, grey dots), or (iii) with a combination of cISD and low dose tTregs (PBMC:tTreg ratio 2:1; CSA/tTreg n=5, blue triangles; MMF/tTreg, blue squares; MP/tTreg n=6, blue dots). Postmortem liver **(A)**, lung **(B)** and spleen **(C)** were analyzed by flow cytometry to quantify the amount of infiltrating CD45+ cells. Depicted are individual values and means ± SEM. Statistical analysis was performed as One-Way-ANOVA with Tukey correction for multiple comparison. Statistical significance levels are indicated as p < 0.05 (dotted line), p < 0.01 (dashed line), p < 0.001 (bold line), p < 0.0001 (solid line with asterix). CSA, Cyclosporine A; MMF, Mycophenolate Mofetil; MP, Methylprednisolone.

**Figure 5:**
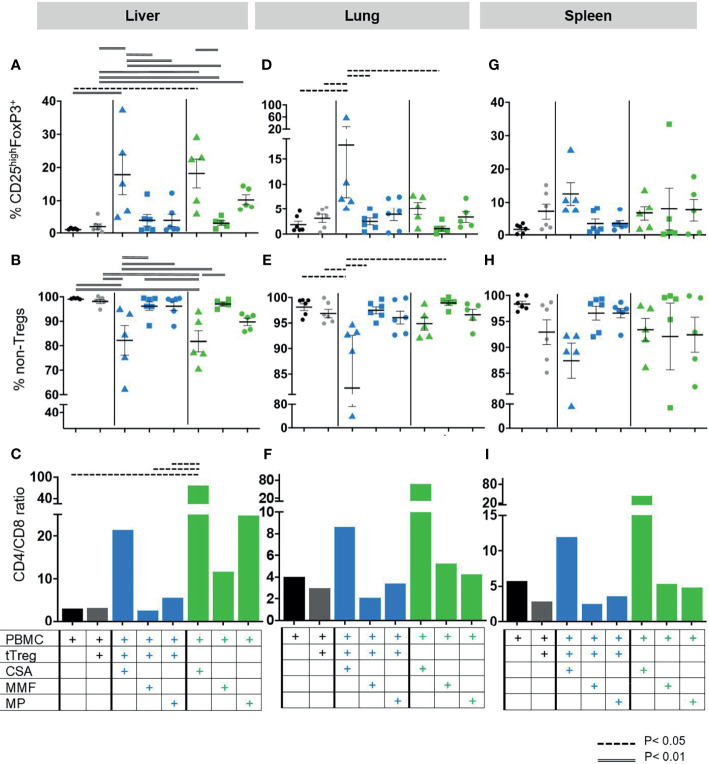
Composition of lymphocytic organ infiltrates. As described in **Figure 1** GvHD was induced by 3x10^6^ human PBMCs in NOD/SCID/IL2Rgamma-/- mice. Mice were subsequently treated with (i) the cISD (green symbols) CSA (4 mg/kg s.c., triangles), MMF (0.5 mg p.o, squares) or MP (20 mg/kg i.p., dots) (n=5) alone (n=5), (ii) with *ex vivo* expanded tTregs at a PBMC:tTreg ratio of 2:1 (n=6, grey dots), or (iii) with a combination of cISD and low dose tTregs (PBMC:tTreg ratio 2:1; CSA/tTreg n=5; MMF/tTreg, n=6; MP/tTreg n=6, blue symbols). Organ samples from liver, lung and spleen were further analyzed by flow cytometry to assess lymphocytic infiltration and proliferation. All samples were analyzed directly after harvesting as outlined in **Table 1**. To this end human live lymphocytes were identified as human CD45+ cells and subsequently gated as CD3+ cells. CD3+ T-lymphocytes were subdivided into CD4+ and CD4- cells. Treg cells were identified as CD25highFoxP3+ cells within the CD4+ compartment **(A, D, G)**, while the remaining CD4+ cells were considered as non-Treg CD4+ T-cells **(B, E, H)**, CD8^+^ T-cells were identified as CD3^+^CD4^-^. Subsequently CD4/CD8 ratios were analyzed for each group. **(C, F, I)**. Depicted are individual values and mean values ± SEM. Statistical analysis was performed as One-Way-ANOVA with Tukey correction for multiple comparisons. Statistical significance levels are indicated as p < 0.05 (dashed line), p < 0.01 (doubled line), p < 0.001 (solid line). CSA, Cyclosporine A; MMF, Mycophenolate Mofetil; MP, Methylprednisolone.

The superior efficacy of CSA and CSA/Treg co-administration as compared to the alternative treatment regimen tested became clearer when cellular infiltrates were analyzed in more detail (**Figures 4**, **5**), yielding statistically significant differences.

To more accurately quantify cellular infiltration and further characterize the phenotype of lymphocytic infiltrates, liver (**Figure 4A**), lung (**Figure 4B**) and spleen (**Figure 4C**) tissues were analyzed by flow cytometry. In line with clinical experience, which classifies the liver as primary target organ of GvHD, we found massive infiltration by human CD45+ leukocytes in the liver (**Figure 4A**, black dots), but also in lungs (**Figure 4B**, black dots) and the spleen (**Figure 4C**, black dots). The mildly increased frequency of human leukocytes in case of low-dose Treg monotherapy, reflected the increased amount of circulating human cells but failed to reduce the frequency of infiltrating cells (**Figures 4A–C**, grey dots). Addition of CSA to Treg therapy significantly reduced organ infiltration in the liver, lung and spleen (**Figures 4A–C**, blue triangles). In contrast, neither tTreg/MMF (**Figures 4A–C** blue squares) nor tTreg/MP (**Figures 4A–C**, blue dots) co-administration was sufficient to reduce the hepatic, pulmonary or splenic infiltration of human leukocytes as compared to the untreated PBMC control (**Figures 4A–C**, black dots). Reassuringly, if only tTregs but no PBMCs were administered we did not observe infiltration of human cells into any organ (data not shown).

### tTreg Recruitment to GvHD Target Tissues Is Promoted by CSA Co-Administration

As PBMC induced GvHD resulted in high frequencies of human leukocytes in the liver, lungs, and spleen, we further aimed to differentiate the composition of leukocytic organ infiltrates and assess the effect of cISD on tTregs in samples from livers (**Figures 5A–C**), lungs (**Figures 5D–F**) and spleens (**Figures 5G–I**). To this end these organ samples were analyzed for the specific frequency of CD4^+^CD25^high^FoxP3^+^ tTreg (**Figures 5A, D, G**) and CD4+ non-Tregs (**Figures 5B, E, H**). Additionally, the CD4/CD8 ratio was assessed in all samples (**Figures 5C, F, I**). Cellular infiltrates in all organs consisted of mainly CD3^+^CD4^+^ non-Treg lymphocytes (**Figures 5B, E, H**). In line with our clinical observations, co-administration of CSA and Tregs led to strong recruitment of circulating CD4^+^CD25^high^FoxP3^+^ Tregs to liver, lungs and spleens (**Figures 5A, D, G**) and substantially reduced the frequency of CD3^+^CD4^+^ non-Treg lymphocytes in these organs (**Figures 5B, E, H**). This effect was superior to any other therapeutic approach in lungs and spleens **Figures 5D, E, G, H**) and, in hepatic tissues, co-administration of CSA and Tregs was comparable to CSA monotherapy (**Figures 5A, B**). In line with the survival data presented in **Figure 2**, co-administration of tTregs and MP (**Figures 5A, G**) substantially reduced the hepatic and splenic proportion of tTregs as compared to MP monotherapy (**Figures 5A, G**). Similarly, combined administration of Treg and CSA reduced the frequency of infiltrating CD8 T-cells as expressed by increased CD4/CD8 ratios in these organs and was with this respect superior to both Treg/MMF and Treg/MP therapy (**Figures 5C, F, I**).

## Discussion

The main focus of this study was to assess if any of the cISD used in first and second line GvHD prophylaxis or therapy either hamper or support the immunomodulatory functions of adoptively transferred tTregs *in vivo*. To this end we employed a humanized GvHD mouse model and found that *in vivo* survival and function of adoptively transferred tTregs are supported by combining them with CSA therapy, leading to significant reduction of harmful lymphocytic organ infiltration and limited clinical progression of GvHD. Similarly, the clinical course of mice treated with MMF and low-dose tTregs significantly improved. Conversely, we found that co-administration of steroids and tTregs negatively impacts the histological state in the studied tissues and the clinical outcome of GvHD.

During acute GvHD, glucocorticoids are usually administered at high doses to control allo-specific immune responses and tapered thereafter to the minimal dose required to prevent GvHD recurrence. The mechanism of action is mediated by DNA-binding steroid-receptor complexes that form in the cytosol and translocate to the nucleus where they directly interfere with the transcription factors AP-1 and NFkB (64, 65). In-depth data on the direct effects of corticosteroids on tTregs are lacking. Previous studies have shown that corticosteroids increase the frequency of circulating Tregs (51–53), and may amplify expansion of Tregs in an IL-2 (54, 55) or dendritic cell (66–70) dependent manner. These data however were not consistently confirmed (56) and we can only speculate on whether the *ex vivo* manipulation and expansion protocol of tTregs or the setting of GvHD might be responsible for the observed differences. Another reason for the aforementioned discrepancies might be the imprecise definition of Tregs, with some groups including induced Tregs in their analysis, while others used additional markers such as CD127, CD45RA and HLA-DR. However, steroid-induced FoxP3 expression (71) seems to not correlate with suppressive activity of these cells (72), and after recruitment to an inflamed tissue environment, these cells might exert effector T-cell function and promote a corticosteroid-dependent condition in patients suffering from GvHD. Last but not least, glucocorticoid-induced T-cell apoptosis is well described on a functional level, but is not well understood on a molecular level (73, 74). In light of the detrimental effect of MP on the clinical outcome we would strongly suggest minimizing the use of glucocorticoids in the case of tTreg therapy.

MMF is typically used in combination with prednisone and CSA. MMF is converted to its pharmacologically active form, Mycophenolic acid (MPA), by enteric and hepatic bioactivation. MPA inhibits proliferation of B- and T-lymphocytes primarily *via* the enzyme inosine monophosphate dehydrogenase, which is crucial for *de novo* purine synthesis. Substantial data on the specific effects of MPA on tTreg function and survival are insufficient as, in most studies, MMF was combined with CSA or steroids, but according to prevailing opinion MMF is compatible with adoptive tTreg transfer (43, 57, 75). A beneficial effect of MMF was postulated by the observation that the amount of circulating tTregs is significantly higher in kidney transplant patients treated with a combined therapy consisting of CSA, steroids and MMF as compared to patients who received CSA, steroids and Everolimus; however, it should be to taken into account that in this study CSA trough levels were significantly higher in the MMF treated group (46). In conclusion, we present, for the first time, data that indicates MMF combined with adoptively transferred tTregs may work synergistically to ameliorate the clinical signs of GvHD, which was reflected in histologically reduced cellular infiltration in the lung and liver. We were, however, unable to quantitatively confirm the additive effect of MMF and tTregs on a cellular level by organ flow cytometry, most likely due to the technical limitations of small organ anatomy and the overall limited cell number yield during organ preparation.

Calcineurin inhibitors, in particular CSA, are one of the most commonly used immunosuppressive compounds for maintenance immunosuppression in transplantation medicine, irrespective of the transplanted organ. By inhibition of the intracellular phosphatase calcineurin, cytosolic activation and nuclear translocation of the nuclear factor of activated T-cells is reduced, thereby hampering *de novo* synthesis of proinflammatory cytokines such as IL-2. Accordingly, the current paradigm holds that under most circumstances CSA leads to reduced *de novo* conversion of allospecific Tregs (45), reduced frequency of circulating tTregs (46), and reduced suppressive capacities (47). However, these results were generated in the clinical setting of solid organ or hematopoietic transplantation where cISD are administered at high doses. Therefore, conceptually different approaches were necessary to recognize that low-dose CSA can increase the frequency of circulating tTregs and promote tTreg function and survival (47, 76–78). In line with these findings, we previously reported that tTreg therapy can replace permanent pharmacologic immunosuppression only if tTreg transfer was combined with early-phase, short-term and low-dose CNI treatment (20). Within a Phase I/IIa clinical trial, we were able to further demonstrate that autologous Treg therapy allows tapering of standard triple immunosuppression in kidney transplanted patients to CNI (Tacrolimus) monotherapy if Tregs are administered early after kidney transplantation (79). The mechanism behind this was previously elucidated by Ruppert and colleagues, who showed that Tregs resist CSA-induced cell death *via* CD44-mediated signaling pathways in a dose-dependent fashion (39). In contrast to our data presented here, Zeiser et al., suggested that coadministration of CSA and Tregs hampers the suppressive capacity of tTregs (57). The authors further describe that the combination of CSA and tTregs strongly reduced survival in a GvHD mouse model, whereas this was not the case when tTregs were combined with MMF. However, the model employed by Zeiser et al., does not recapitulate the clinical state where we face either ongoing GvHD or the post-transplant phase with a particularly high risk of clinically relevant allo-responses (57). Additionally, Zeiser et al., administered 10 mg/kg CSA while in the model presented here, we use only 4 mg/kg body weight, which more closely corresponds to the clinical dosage in human patients (20, 58, 59). Therefore, it is perhaps unsurprising that in our present study we were able to confirm the beneficial effect of CSA and tTreg co-administration.

In summary, we have demonstrated that the efficacy of *ex vivo* pre-activated tTregs to control GvHD reactions by alloreactive T-cells is increased when combined with the calcineurin inhibitor CSA and, to a lesser extent, when combined with MMF. In contrast, the combination of tTregs with steroids in this context reduced tTreg treatment efficacy. In our mouse model, GvHD is due to clonal expansion of naïve xenoreactive T-cells and their differentiation to pathogenic effector T-cells. The data support synergistic inhibitory effects of CSA and tTreg on this process, whilst the functionality of pre-activated tTregs used for adoptive transfer appears to be resistant to CSA. The model presented here has one limitation: *in vivo* expansion of human tTreg in the humanized mouse graft is inherently poor, therefore, a relatively high tTreg/Teff ratio (*e.g.* >1:2) is necessary to see beneficial effects, confirming findings from numerous other groups (80–83). Thus, the use of CSA supports tTreg activity, but in a xenogenic mouse model high cell doses are necessary to compensate for poor tTreg clonal expansion. To date, the numbers of adoptively transferred tTregs required to obtain beneficial effects in a human context remains unclear. However, we conclude that CSA should be the agent of choice when adoptive transfer of a sufficient number of tTregs for GvHD is scheduled. MMF may be a further beneficial combination partner if dual or alternative therapy is required. Steroids should be avoided or, if necessary, be restricted to minimal doses.

## Data Availability Statement

The raw data supporting the conclusions of this article will be made available by the authors, without undue reservation.

## Ethics Statement

The studies involving human participants were reviewed and approved by Charité-Universitätsmedizin Berlin, Ethics Committee (Institutional Review Board), Charitéplatz 1, 10117 Berlin. The patients/participants provided their written informed consent to participate in this study. The animal study was reviewed and approved by Landesamt für Gesundheit und Soziales Berlin (G0483/09), Berlin, Germany.

## Author Contributions

SL-K supervised the project, designed the experiments, performed some experiments, analyzed the data, and wrote the manuscript. AZ, AF, HH, and IS-K performed and analyzed experiments. MS-H contributed to the data analysis and assisted in writing the manuscript. RK performed the histological analysis of solid organs. H-DV and PR led the project, designed the research, assisted in data interpretation, and contributed the writing of the manuscript. All authors contributed to the article and approved the submitted version.

## Funding

This project has received funding from the European Union’s Horizon 2020 Research and Innovation Programme under grant agreement No 825392 (RESHAPE) and from the European Union, Seventh Framework Programme [FP7/2007-2013], under grant agreement n° HEALTH-F5-2010-260687 The ONE Study. The project was further funded by the German Federal Ministry of Education and Research (BMBF) under grant agreement with the Berlin Institute of Health (BIH) and BIH Center for Regenerative Therapies (BCRT). SL-K was supported by a personal “Clinical Scientist” grant (BSRT/BCRT) and the Rahel-Hirsch-Habilitation Grant (Charité). The funders had no role in considering the study design or in the collection, analysis, interpretation of data, writing of the report, or decision to submit the article for publication.

## Conflict of Interest

The authors declare that the research was conducted in the absence of any commercial or financial relationships that could be construed as a potential conflict of interest.

## Publisher’s Note

All claims expressed in this article are solely those of the authors and do not necessarily represent those of their affiliated organizations, or those of the publisher, the editors and the reviewers. Any product that may be evaluated in this article, or claim that may be made by its manufacturer, is not guaranteed or endorsed by the publisher.
