# Supplementary material for: Cyclosporine A but Not Corticosteroids Support Efficacy of Ex Vivo Expanded, Adoptively Transferred Human Tregs in GvHD
Source: Front Immunol. 2021 Oct 11;12:716629. doi: 10.3389/fimmu.2021.716629 (PMC8543016; doi:10.3389/fimmu.2021.716629)
Supplement: Supplementary file 1 [file DataSheet_1.pdf]

Supplementary Material to

Cyclosporine A but Not Corticosteroids Support Efficacy of Ex Vivo Expanded, Adoptively Transferred Human Tregs in GvHD

Sybille Landwehr-Kenzel [1,2,3*], Anne Zobel [2], Isabela Schmitt-Knosalla [4], Anne Forke [2], Henrike Hoffmann [2], Michael Schmueck-Henneresse [2,3], Robert Klopfleisch [5], Hans-Dieter Volk [2,3,4] , and Petra Reinke [2,3,6]

[1] Department of Pediatric Respiratory Medicine, Immunology and Critical Care Medicine, Charité – Universitätsmedizin Berlin, Corporate Member of Freie Universität Berlin and Humboldt-Universität zu Berlin, Berlin, Germany, [2] Berlin Institute of Health (BIH) at Charité – Universitätsmedizin Berlin, BIH-Center for Regenerative Therapies (BCRT), Berlin, Germany, [3] Charité - Universitätsmedizin Berlin, Corporate Member of Freie Universität Berlin and Humboldt-Universität zu Berlin, Berlin Center for Advanced Therapies (BeCAT), Berlin, Germany, [4] Charité – Universitätsmedizin Berlin, Corporate Member of Freie Universität Berlin and Humboldt-Universität zu Berlin, Institute of Medical Immunology, Berlin, Germany, [5] Department of Veterinary Medicine, Institute of Veterinary Pathology, Freie Universität Berlin, Berlin, Germany, [6] Department of Nephrology and Internal Intensive Care Medicine, Charité – Universitätsmedizin Berlin, Corporate Member of Freie Universität Berlin and Humboldt-Universität zu Berlin, Berlin, Germany.

Supplementary Table ST1: GvHD scoring based on clinical signs.

Clinical Score	Clinical status
0	no GvHD: clinically healthy
1	mild GvHD: weight loss < 10%, mild pallor, slightly scurfy fur.
1.5	mild GvHD: weight loss < 10%, mild pallor, slightly scurfy fur, streaky tail
2	moderate GvHD: weight loss 10-15 %, pale, scurfy fur, streaky tail, hunched back
2.5	moderate GvHD: weight loss 10-15 %, pale, scurfy fur, streaky tail, hunched back, reduced mobility
3	severe GvHD: weight loss 15-20% plus strongly hunched back, minimal mobility or immobile

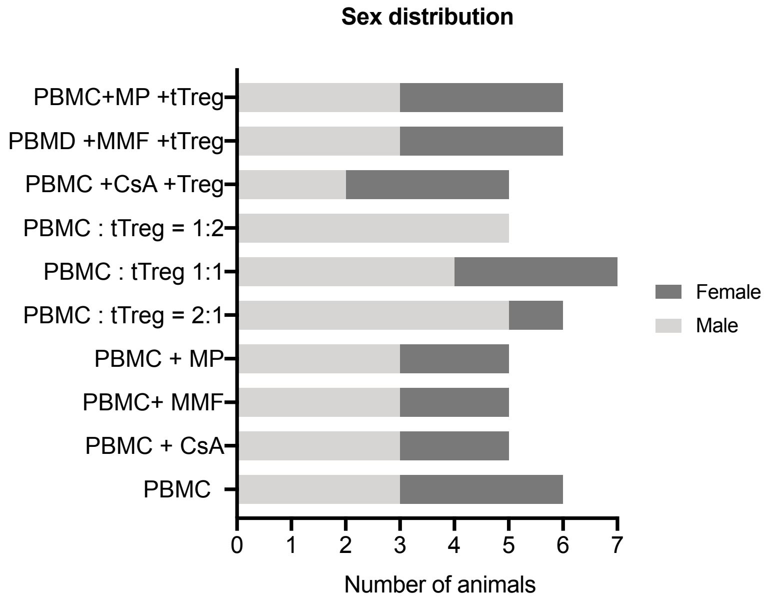

Supplementary Figure S1: Sex distribution of animals. All animals used were adult male and female Balb/c NOD/SCID IL-2Rgamma-/- mice. We aimed for equal distribution of male and female animals to all groups. Depicted are numbers of male and female animals for all individual treatment groups.

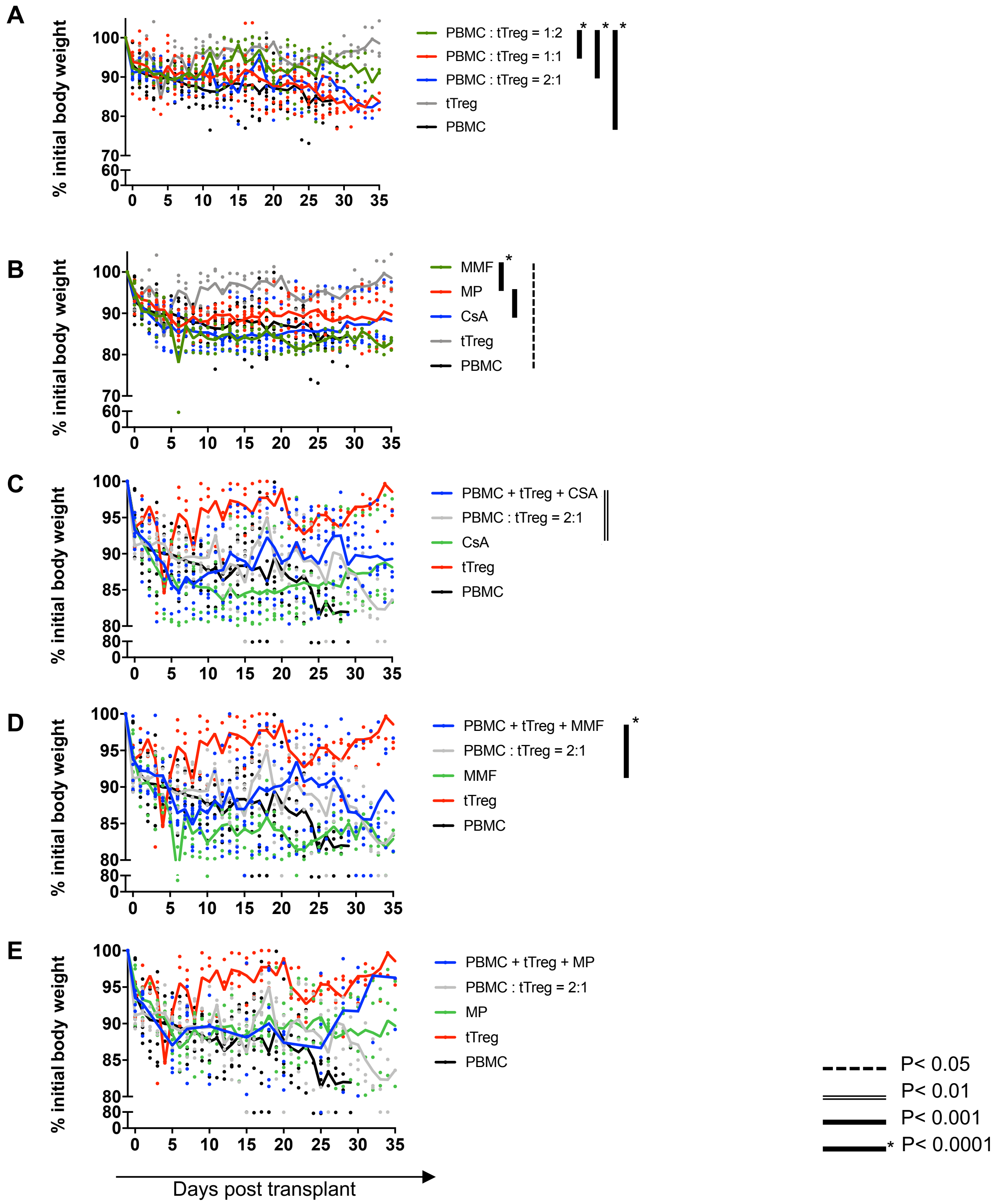

Supplementary Figure S2: Clinical course and survival rates. As described in Fig. 1 GvHD was induced in NOD/SCID/IL-2Rgamma -/- mice by infusion of $3x10^6$ human PBMC (GvHD control, **A – E**). tTreg control mice received no PBMCs but $3x10^6$ *ex vivo* expanded human tTregs alone (tTreg control, **A – E**). **(A)** In parallel to GvHD induction with PBMCs, mice were treated with escalating doses of *ex vivo* expanded tTregs at a ratio of 2:1 (n=6), 1:1 (n=7) or 1:2 (n=5). **(B)** Alternatively, mice were treated with the cISD **(C)** CSA (4 mg/kg s.c.), **(D)** MMF (0.5 mg p. o.) or **(E)** MP (20 mg/kg i.p.) (each group n=5) alone or **(C-E)** in combination with tTreg (PBMC:tTreg ration 2:1). Statistical analysis was performed as One-Way-ANOVA with Tukey correction for multiple comparison. Statistical significance levels are indicated as $p < 0.05$, $p < 0.01$, $p < 0.001$, $p < 0.0001$. CSA: Cyclosporine A, MMF: Mycophenolate Mofetil, MP: Methylprednisolone.

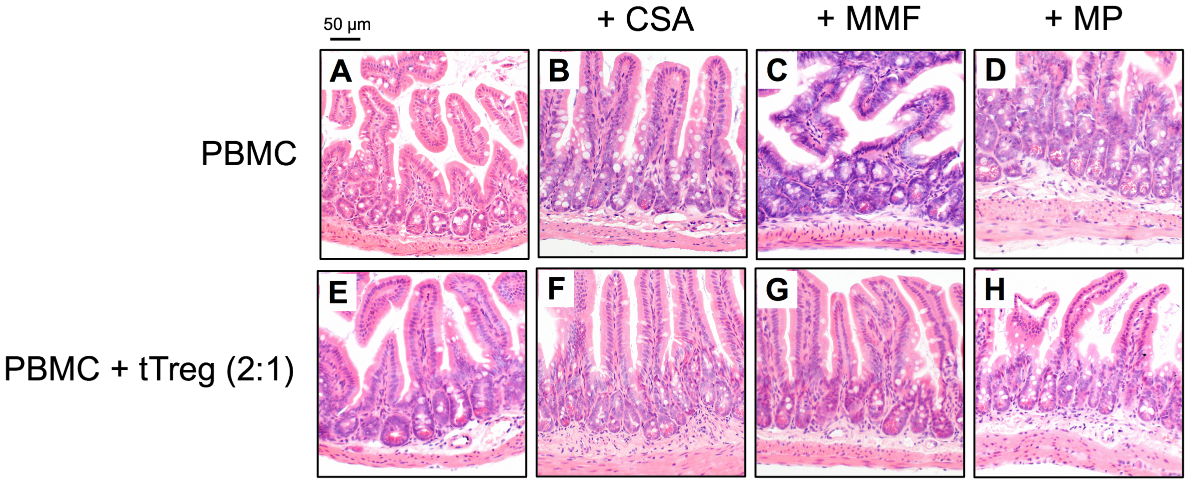

Supplementary Figure S3: Histopathological findings of the intestinal tract. Intestinal samples of all mice were stained by hematoxylin and eosin (H&E). No signs of GvHD such as cellular infiltration, apoptotic epithelia cells or changes in the tissue architecture were detected. Shown are representative photographs from intestinal samples of mice which received **(A)** PBMCs only, PBMCs plus one of the cISD **(B)** CSA, **(C)** MMF, **(D)** MP, **(E)** PBMCs plus tTregs or PBMC and coadministration of **(F)** CSA/tTreg, **(G)** MMF/tTregs or **(H)** MP/tTregs.
